# QT and Tpeak‐Tend interval variability: Predictive electrical markers of hospital stay length and mortality in acute decompensated heart failure. Preliminary data

**DOI:** 10.1002/clc.23888

**Published:** 2022-09-09

**Authors:** Gianfranco Piccirillo, Federica Moscucci, Myriam Carnovale, Gaetano Bertani, Ilaria Lospinuso, Ilaria Di Diego, Andrea Corrao, Teresa Sabatino, Giulia Zaccagnini, Davide Crapanzano, Pietro Rossi, Damiano Magrì

**Affiliations:** ^1^ Department of Clinical and Internal Medicine, Anesthesiology and Cardiovascular Sciences, Policlinico Umberto I “Sapienza” University of Rome Rome Italy; ^2^ Cardiology Division Arrhythmology Unit, S. Giovanni Calibita, Isola Tiberina Rome Italy; ^3^ Department of Clinical and Molecular Medicine, S. Andrea Hospital “Sapienza” University of Rome Rome Italy

**Keywords:** decompensated chronic heart failure, QT, Tpeak‐Tend, QTVI, temporal dispersion of repolarization phase

## Abstract

**Background:**

As previously reported, an increased repolarization temporal imbalance induces a higher risk of total/cardiovascular mortality.

**Hypothesis:**

The aim of this study was to assess if the electrocardiographic short period markers of repolarization temporal dispersion could be predictive of the hospital stay length and mortality in patients with acutely decompensated chronic heart failure (CHF).

**Method:**

Mean, standard deviation (SD), and normalized variance (VN) of QT (QT) and Tpeak‐Tend (Te) were obtained on 5‐min ECG recording in 139 patients hospitalized for acutely decompensated CHF, subgrouping the patients for hospital length of stay (LoS): less or equal 1 week (≤1 W) and those with more than 1 week (>1 W).

**Results:**

We observed an increase of short‐period repolarization variables (TeSD and TeVN, *p* < .05), a decrease of blood pressure (*p* < .05), lower ejection fraction (*p* < .05), and higher plasma level of biomarkers (NT‐proBNP, *p* < .001; Troponin, *p* < .05) in >1 W LoS subjects. 30‐day deceased subjects reported significantly higher levels of QTSD (*p* < .05), Te mean (*p* < .001), TeSD (*p* < .05), QTVN (*p* < .05) in comparison to the survivors. Multivariable Cox regression analysis reported that TeVN was a risk factor for longer hospital stay (hazard ratio: 1.04, 95% confidence limit: 1.01–1.08, *p* < .05); whereas, a longer Te mean was associated with higher mortality risk (hazard ratio: 1.02, 95% confidence limit: 1.01–1.03, *p* < .05).

**Conclusion:**

A longer hospital stay is considered a clinical surrogate of CHF severity, we confirmed this finding. Therefore, these electrical and simple parameters could be used as noninvasive, transmissible, inexpensive markers of CHF severity and mortality.

## 1 INTRODUCTION

The prevalence of chronic heart failure (CHF) is approximately 10% among the over 70 years‐old elderly^1^ and there are 600 000 and 500 000 new cases per year, respectively, in Europe and in the United States.^2^ Therefore, this complex, progressive clinical syndrome frequently causes hospital readmission, high levels of mortality rates, and a huge amount of healthcare costs. Particularly, it was previously observed that the longer the hospital stay, the poorer the prognosis of the patients was.^3^, ^4^, ^5^, ^6^ In some previous studies, in acutely decompensated CHF patients, some noninvasive electrical markers, based on short‐term repolarization temporal dispersion (such as QT, QTe, Te variance normalized [VN]) were found capable of specifically selecting high risk cardiovascular and all‐cause mortality patients, confirming a strict relationship with some biomarkers of decompensated CHF.^7^, ^8^ Predicting the length of hospitalization and the possibility to benefit from the intensive care and specific therapies during the acute phase of his illness could be highly helpful for health‐care systems, to concentrate their energies in terms of personnel and resources. Consequently, the duration and variability of QT and T peak‐end (Te, expressed as standard deviation, SD) intervals as markers of mortality and severity of CHF, expressed as hospital length of stay^3^, ^9^ were analyzed.

## 2 METHODS

### 2.1 Patients and protocol

Consecutive in‐patients were enrolled when admitted to the Department of Geriatric Medicine, from January 2019 to September 2020, due to decompensated CHF in NYHA functional class IV at the enrollment. Patients were defined as decompensated following the European Society of Cardiology guidelines.^1^ Moreover, patients were divided into two groups: the first with an in‐hospital stay length (LoS) less or equal to 1 week (≤1 W) and the second with more than 1 week (>1 W). When enrolled, all patients underwent: clinical history, physical examination, standard electrocardiogram (ECG) and transthoracic echocardiography, 5 min of II lead ECG (MiocardioEvent™) recording, and a blood sample for NT‐proBNP dosage, obtained by Alere Triage Analyzer (Alere) and the other serum variables. The same ECG data were collected at the discharge in a subgroup of survived patients, together with a new NT‐proBNP evaluation, to verify the resolution of the acute decompensation and the reliability of ECG data. The Cockcroft–Gault formula was used to assess creatinine clearance.

All patients provided written informed consent for the use of their records for research purposes and the study was in accordance with good clinical practice and the principles of the Declaration of Helsinki regarding clinical research involving human patients. The study underwent the Ethical Committee of Policlinico Umberto I approbation. The ClinicalTrials.gov number is NCT04127162.

### 2.2 Offline data analysis

Five minutes ECG (Miocardio Event™) II lead signals were acquired, digitalized at a sampling frequency of 500 Hz, and wirelessly transmitted to a cloud platform for data storage via mobile phone^7^, ^8^ Subsequently, all digitized signal recordings were downloaded by the cloud and checked by a single physician (G. P.) blinded to subjects' circumstances. Therefore, we measured the following intervals from the respective time series in ECG recordings: RR, QT, and Te; QT was obtained by measuring the interval from the onset of the Q‐wave to the T‐wave end; Te was obtained from the T peak to the end of T wave. To identify the repolarization intervals, we used software originally proposed by Berger^10^ and validated in other studies.^11^, ^12^, ^13^, ^14^

We, therefore, calculated mean, variance, and SD (QTSD and TeSD) values for each of these repolarization phase intervals and, finally, we calculated the VN for a mean of QT (QTVN) and Te (TeVN)^12^, ^14^, ^15^, ^16^, ^17^:

QTVN = QT variance/(QTe mean)^2^,

TeVN = Te variance/(Te mean)^2^.

Software for data analysis was designed and produced by our research group with the LabView program (National Instruments).

### 2.3 Statistical analysis

All variables with normal distribution were expressed as means ± SD whereas non‐normally distributed variables were as median and interquartile range (i.r.). Categorical variables were analyzed with the *χ* ^2^ test. Unpaired Student's *t*‐test was used to compare data for the normally distributed variables; on the contrary, Mann–Whitney tests were used to compare non‐normally distributed variables (as evaluated by the Kolmogorov–Smirnov test). Uni‐ and multivariable forward (A. Wald) Cox proportion‐hazard regression analysis was used to determine the association between repolarization variables (covariates) and some dichotomized variables (dependent variables). In particular, we considered separately the following dependent variables: first, 30‐day mortality, and, second the different lengths of in‐hospital stay (≤1 W or >1W). *p* values of less than or equal to .05 were considered statistically significant. All data were evaluated by use of the database SPSS‐PC+ (SPSS‐PC+ Inc.).

## 3 RESULTS

Of the initially 146 eligible patients, 6 were excluded because the repolarization signals were suboptimal for the analysis, consequently, 139 patients were studied. At the time of presentation, all patients were in NYHA functional class IV. 29 patients died within 30 days from the enrollment (overall mortality rate 21%): 19 patients died of respiratory failure, 7 from terminal heart failure, 2 from fatal acute myocardial infarction, 1 from arrhythmic sudden cardiac death (sustained ventricular tachycardia and ventricular fibrillation). The demographic and clinical characteristics of the two groups are provided in Table 1. As abovementioned, the subjects were grouped according to the in‐hospital LoS and the ≤1 W of LoS group showed significantly higher levels of blood pressure (*p* < .01), better left ventricular ejection fraction (*p* < .05), in range blood calcium concentration (*p* < .001) than >1 W of LoS one (Table 1). In addition, the same group reported significantly lower levels of NT‐pro BNP (*p* < .001) and lower high‐sensitivity cardiac troponin T (*p* < .05) (Table 1). Finally, patients in the >1 W of LoS group were more prone to show valves' diseases (*p* < .05).

**Table 1:** Characteristics of the study subjects

	All subjects	Length of hospital stay ≤ week subjects	Length of hospital stay > week subjects	*p*
	**N:139**	**N:52**	**N:87**	
Hospital length of stay, days	12 [15]	3 [5]	18 [13]	‐
Age, years	83 ± 10	84 ± 9	83 ± 11	.613
M/F, *n*	77/62	25/27	52/35	.180
BMI, kg/m^2^	26 ± 5	26 ± 5	26 ± 5	.720
SBP, mm Hg	126 ± 20	**132 ± 19**	**123 ± 20**	**.007**
DBP, mm Hg	69 ± 11	**73 ± 12**	**66 ± 9**	**<.001**
Heart rate, b/m	72 ± 13	69 ± 12	73 ± 14	.054
Left ventricular ejection fraction, %	43 ± 10	**45 ± 9**	**41 ± 11**	**<.011**
NT‐pro BNP, pg/ml	2160 [6454]	**1010 [2599]**	**2900 [8655]**	**<.001**
High sensitivity cardiac troponin T (ng/L)	36 [41]	**32 [31]**	**122 [51]**	**.019**
C‐protein reaction (mg/dl)	6.5 [7.3]	3.1 [9.2]	2.4 [5.0]	.129
Potassium, mmol/L	4.1 ± 0.3	4.2 ± 0.6	4.1 ± 0.6	.348
Calcium, mmol/L	2.15 ± 0.27	**2.54 ± 0.18**	**2.11 ± 0.27**	**.001**
Creatinine clearance, ml/m	48 ± 28	48 ± 28	47 ± 26	.906
CHF with depressed systolic function, *n* (%)	86 (62)	**26 (50)**	**60 (69)**	**.026**
CHF with preserved systolic function, *n* (%)	53 (38)	**26 (50)**	**27 (31)**	**.026**
Hypertension, *n* (%)	106 (76)	41 (79)	65 (75)	.579
Hypercholesterolemia, *n* (%)	62 (45)	25 (48)	37 (43)	.524
Diabetes, *n* (%)	54 (39)	23 (44)	36 (31)	.314
Renal insufficiency, *n* (%)	65 (47)	19 (37)	46 (53)	.062
Known myocardial ischemia history, *n* (%)	46 (33)	14 (27)	32 (37)	.232
Valve diseases	26 (19)	**5 (10)**	**21 (24)**	**.034**
Premature supraventricular complexes, *n* (%)	17 (12)	6 (12)	11 (13)	.847
Premature ventricular complexes, *n* (%)	30 (22)	10 (19)	20 (14)	.602
Permanent atrial fibrillation, *n* (%)	47 (34)	19 (37)	28 (32)	.599
Left bundle branch block, *n* (%)	27 (19)	9 (17)	18 (13)	.626
Right bundle branch block, *n* (%)	27 (19)	9 (17)	18 (13)	.626
Pacemaker—ICD, *n* (%)	33 (24)	15 (29)	18 (21)	.274
30‐day deceased subjects, *n*	29 (21)	8 (15)	21 (24)	.219
β‐blockers, *n* (%)	89 (64)	34 (65)	55 (63)	.797
Furosemide, *n* (%)	107 (77)	37 (71)	70 (81)	.207
ACE/sartans	60 (43)	22 (42)	38 (44)	.875
Aldosterone antagonists, *n* (%)	17 (12)	5 (10)	12 (14)	.467
Potassium, *n* (%)	7 (5)	3 (6)	4 (5)	.760
Nitrates, *n* (%)	16 (12)	7 (14)	9 (10)	.577
Ivabradine, *n* (%)	5 (4)	2 (4)	3 (3)	.903
Digoxin, *n* (%)	7 (5)	2 (4)	5 (6)	.620
Statins, *n* (%)	43 (31)	21 (37)	22 (25)	.062
Antiplatelet drugs, *n* (%)	51 (37)	24 (46)	27 (31)	.074
Oral anticoagulants, *n* (%)	37 (27)	14 (27)	23 (26)	.950
Diltiazem or verapamil, *n* (%)	6 (4)	2 (4)	4 (5)	.833
Dihydropyridine calcium channel blockers, *n* (%)	18 (13)	10 (19)	44 (9)	.088
Propafenone, *n* (%)	1 (1)	(12)	0 (0)	.194
Amiodarone, *n* (%)	15 (11)	4 (8)	11 (13)	.363
Ranolazina, *n* (%)	5 (4)	0 (0)	5 (6)	.078
Sacubitril/valsartan, *n* (%)	2 (1)	1 (2)	1 (1)	.711

*Note*: Comparison data between patients with different lengths of hospital stay. Data are expressed as mean ± SD, or median [interquartile range], or number of patients (%). Bold values indicate statistically significant.

Subjects from the >1 W LoS group showed a higher TeSD (*p* < .05) and TeVN (*p* < .05) than the ≤1 W (Table 2). In addition, the 30‐day deceased subjects had higher levels of QTSD (*p* < .05), Te mean (*p* < .001), TeSD (*p* < .05), QTVN (*p* < .05) than the survivors' group (Table 3).

**Table 2:** Short period repolarization temporal dispersion variables and hospitalization length of stay

	All	Length of Hospital stay ≤ 1 week subjects	Length of hospital stay > 1 week subjects	*p*
	**N:139**	**N:52**	**N:87**	
QT mean, ms	436 ± 67	444 ± 61	437 ± 74	.610
QT SD, ms	7 [6]	5 [5]	7 [5]	.241
Te mean, ms	104 ± 22	104 ± 20	107 ± 25	.612
Te SD, ms	8 [3]	**6 [4]**	**7 [4]**	**.031**
QTVN	0.20 [0.31]	0.20 [0.33]	0.24 [0.31]	.254
TeVN	4 [6]	**4 [5]**	**5 [7]**	**.045**

*Note*: Comparison data between patients with different lengths of hospital stay. Data are expressed as mean ± SD, or median [interquartile range]. Bold values indicate statistically significant.

**Table 3:** Short period repolarization temporal dispersion variables and mortality

	All	30‐day deceased subjects	Survivors	
	**N**:139	**N:29**	**N:110**	***p***
QT mean, ms	436 ± 67	442 ± 77	434 ± 65	.604
QT SD, ms	7 [6]	**8 [4]**	**5 [5]**	**.005**
Te mean, ms	104 ± 22	**117 ± 30**	**101 ± 19**	**<.001**
Te SD, ms	8 [3]	**8 [3]**	**6 [4]**	**.023**
QTVN	0.20 [0.31]	**0.29 [0.29]**	**0.18 [0.32]**	**.009**
TeVN	4 [6]	4 [5]	4 [6]	.380

*Note*: Comparison data between 30‐day deceased subjects and survivors. Data are expressed as mean ± SD, or median [interquartile range]. Bold values indicate statistically significant.

Uni‐multivariable Cox regression analysis reported just one significant relationship between a longer hospital stay and TeVN (hazard ratio: 1.04, 95% confidence limit: 1.01–1.08, *p* < .05). The same two statistical analyses demonstrated that 30‐day mortality was related to QTSD (*p* < .05), Te mean (*p* < .05) and TeSD (*p* < .05) values (Table 4); nevertheless, the multivariable analysis only selected the 30‐day mortality and the Te mean (*p* < .05) relation (Table 4). Considering the group >1 W apart, the multivariable regression analysis confirmed that mortality was related to the Te mean: *χ* ^2^: 8.56, hazard ratio (95% CI): 1.02 (1.01–1.03), *p*: .003.

**Table 4:** Prediction of mortality in all study patients by Cox regression

Variables	*χ* ^2^	Univariable analysis hazard ratio (95% CI)	*p* Values	*χ* ^2^	Multivariable analysis hazard ratio (95% CI)	*p* Values
QT mean	‐	‐	.644	‐	‐	ns
QT SD	**6.97**	**1.10 (1.03–1.18)**	**.008**	‐	‐	ns
Te mean	**8.56**	**1.02 (1.01–1.03)**	**.003**	**8.56**	**1.02 (1.01–1.03)**	**.003**
Te SD	**5.51**	**1.12 (1.02–1.23)**	**.019**	‐	‐	ns
QTVN	‐	‐	.124	‐	‐	ns
TeVN	‐	‐	.508	‐	‐	ns

*Note*: Data are presented as hazard ratio (95% confidence limit). Bold values indicate statistically significant.

Finally, 65 patients among the 110 survivors were re‐evaluated before discharge (ECG markers and NT‐pro‐BNP determination). Neither QT mean (discharge 438 ± 84 ms vs. enrollment 447 ± 79, *p*: .31), nor Te mean (discharge 105 ± 28 ms vs. enrollment 105 ± 30; *p*: .6) at discharge showed substantial changes compared to the hospital admission. Conversely, the blood concentration of NT‐pro‐BNP was found to be lower at discharge than at admission (1900 pg/ml, i.r. 3287 vs. 2910 pg/ml, i.r. 7080, *p* < .0001), as expected.

## 4 DISCUSSION

The major new finding of the present work was that some simple, noninvasive, inexpensive, easily repeatable electrical markers, based on short‐term repolarization variability, were associated with the in‐hospital LoS and mortality. In particular, TeVN and Te mean were respectively predictive of the LoS and 30‐day mortality, when obtained at hospital admission. In‐hospital LoS was considered a marker of severity of decompensated CHF.^3^, ^9^, ^18^ Although, in fact, the mortality prevalence in our study was similar among the two studied groups, whereas, in the >1 W LoS group the major part of low ejection fraction and impaired blood pressure, longer repolarization temporal dispersion, higher level of CHF biomarkers (NT‐proBNP and troponin) cases were found. In other words, clinical heart failure severity was connected to the in‐hospital LoS.

The pathophysiological basis of Te segment prolongation is controversial; while Te temporal dispersion (TeVN) is still little studied, some authors believe that this interval represents a noninvasive marker of transmural dispersion of repolarization in the left ventricle,^19^ but other authors oppose this interpretation without offering alternative explanations.^20^ If the pathophysiologic basis of the Te is not clear, the clinical use of this marker was not disputable. In fact, a recent meta‐analysis by Tse and coll,^21^ involving 155 856 subjects with different heart diseases, reported an increased risk of total mortality, sudden cardiac death, and cardiovascular mortality associated with prolonged Te. Lately, we observed in decompensated severe CHF that Te means duration was related not only to mortality but even to NT‐proBNP concentrations.^7^, ^8^ Even without observing a substantial difference in Te mean among <1 W and ≥1 W groups of patients, in the present study, an increase in temporal dispersion of the same sub‐interval (TeSD and TeVN) was observed. The possible reasons for this repolarization inhomogeneity are probably the different severity of CHF. In fact, sodium, calcium, and potassium channels and transport mechanisms are upset in CHF^22^ and the final result is a prolonged temporal dispersion of QT and Te.^14^, ^15^, ^16^, ^17^ Recent observations have highlighted that sympathetic hyperactivity is able to induce an increase in QTe variability index, QTeVN, TeVN, TeSD, in normal^17^ and pathological^23^, ^24^ conditions.

Thus, the morphological and structural alterations of the myocardium resulting in specific modifications of these electrocardiographic markers. This allows for the patient's telemonitoring over time, as when the clinical and morpho‐structural improvement occurs (no more swelling nor alteration of the functionality of the ion channels), a relative improvement of the electrocardiographic parameters should be observed.

In conclusion, patients with acute decompensated CHF and ≥1 W length of in‐hospital stay showed elevated short‐term repolarization markers. In particular, these patients had higher TeVN, TeSD, and higher levels of CHF biomarkers.

Finally, in the subgroup of re‐evaluated, survived patients, the substantial stability of both QT mean and Te mean from admission to discharge suggests, once again, the predictive capability of these ECG markers. As shown in Table 3, Te mean showed a noticeable difference in the hospital admission between survived and deceased patients; therefore, Te mean tends to be able to identify patients with worse prognoses.

Obviously, the present study supplies a working hypothesis, that needed to be confirmed in larger and case–control studies.

Nevertheless, it could be hypothesized that these markers could be used as a noninvasive, transmissible, inexpensive, electric, surrogate marker of hospital length of stay, of CHF clinical severity, suggesting higher mortality risk.

## 5 LIMITATIONS

The main limitation of this study is the small sample size. This aspect can be overcome by continuing the enrollment and the involvement of more centers. With an adequate sample size, it will be possible to verify the predictive power of the variables under examination even in subjects with less severe degrees of decompensated CHF.

In addition, an actual limitation of the study is the absence of patients treated with SGLT2 inhibitors. The sample was in fact studied before the recent indications provided by the European Society of Cardiology guidelines on the use in class I evidence A of these drugs in subjects with heart failure.^25^ The use of these drugs could in fact have some impact on the clinical presentation and duration of hospitalizations. It will be the authors' responsibility to deepen this data in a further study.

Furthermore, the geographic variability, already described in the past,^26^ in the duration of hospitalization and rehospitalization of patients suffering from heart failure is a relevant datum. In this study, carried out in a single center, it is not possible to highlight discrepancies from this point of view. The involvement of further centers could ensure a better definition of the geographical impact.

## CONFLICT OF INTEREST

The authors declare no conflict of interest.

## Data Availability

Data could be provided on request to the corresponding author.

## References

[1] Piotr P , Adriaan AV , Stefan DA , et al. ESC Guidelines for the diagnosis and treatment of acute and chronic heart failure: the Task Force for the diagnosis and treatment of acute and chronic heart failure of the European Society of Cardiology (ESC) Developed with the special contribution of the Heart Failure Association (HFA) of the ESC. Eur Heart J. 2016;37(27):2129‐2200. 27206819 10.1093/eurheartj/ehw128

[2] Dokainish H , Teo K , Zhu J , et al. Global mortality variations in patients with heart failure: results from the International Congestive Heart Failure (INTER‐CHF) prospective cohort study. Lancet Glob Health. 2017;5(7):e665‐e672. 28476564 10.1016/S2214-109X(17)30196-1

[3] Kristi R , Melissa GB , Teresa M , et al. Relation of acute heart failure hospital length of stay to subsequent readmission and all‐cause mortality. Am J Cardiol. 2015;116(3):400‐405. 26037295 10.1016/j.amjcard.2015.04.052

[4] Zaya M . Predictors of re‐hospitalization in patients with chronic heart failure. World J Cardiol. 2012;4(2):23‐30. 22379534 10.4330/wjc.v4.i2.23 PMC3289890

[5] Tsuchihashi1 M , Tsutsui H , Kodama K , et al. Medical and socioenvironmental predictors of hospital readmission in patients with congestive heart failure. Am Heart J. 2001;142(4):E7. 11579371 10.1067/mhj.2001.117964

[6] Solomon SD , Dobson J , Pocock S , et al. Influence of nonfatal hospitalization for heart failure on subsequent mortality in patients with chronic heart failure. Circulation. 2007;116(13):1482‐1487. 17724259 10.1161/CIRCULATIONAHA.107.696906

[7] Piccirillo G , Moscucci F , Mariani MV , et al. Hospital mortality in decompensated heart failure. A pilot study. J Electrocardiol. 2020;61:147‐152. 32629315 10.1016/j.jelectrocard.2020.05.006

[8] Piccirillo G , Moscucci F , Bertani G , et al. Short‐period temporal dispersion repolarization markers predict 30‐days mortality in decompensated heart failure. J Clin Med. 2020;9(6):1879. 10.3390/jcm9061879 32560151 PMC7356287

[9] Maneesh S , Bing Y , Harindra CW , et al. Associations between short or long length of stay and 30‐Day readmission and mortality in hospitalized patients with heart failure. JACC Heart Fail. 2017;5(8):578‐588. 28501521 10.1016/j.jchf.2017.03.012

[10] Berger RD , Kasper EK , Baughman KL , Marban E , Calkins H , Tomaselli GF . Beat‐to‐beat QT interval variability: novel evidence for repolarization lability in ischemic and nonischemic dilated cardiomyopathy. Circulation. 1997;96(5):1557‐1565. 9315547 10.1161/01.cir.96.5.1557

[11] Piccirillo G , Magrì D , Matera S , et al. QT variability strongly predicts sudden cardiac death in asymptomatic subjects with mild or moderate left ventricular systolic dysfunction: a prospective study. Eur Heart J. 2007;28(11):1344‐1350. 17101636 10.1093/eurheartj/ehl367

[12] Piccirillo G , Magnanti M , Matera S , et al. Age and QT variability index during free breathing, controlled breathing and tilt in patients with chronic heart failure and healthy control subjects. Transl Res. 2006;148(2):72‐78. 16890147 10.1016/j.trsl.2006.02.001

[13] Piccirillo G , Moscucci F , Fabietti M , et al. Age, gender and drug therapy influences on Tpeak‐tend interval and on electrical risk score. J Electrocardiol. 2020;59:88‐92. 32023499 10.1016/j.jelectrocard.2020.01.009

[14] Piccirillo G , Moscucci F , Fabietti M , et al. Arrhythmic risk in elderly patients candidates to transcatheter aortic valve replacement: predictive role of repolarization temporal dispersion. Front Physiol. 2019;10:991. 31447689 10.3389/fphys.2019.00991 PMC6691061

[15] Baumert M , Porta A , Vos MA , et al. QT interval variability in body surface ECG: measurement, physiological basis, and clinical value: position statement and consensus guidance endorsed by the European Heart Rhythm Association jointly with the ESC Working Group on Cardiac Cellular Electrophysiology. Europace. 2016;18(6):925‐944. 26823389 10.1093/europace/euv405 PMC4905605

[16] Piccirillo G , Moscucci F , D'Alessandro G , et al. Myocardial repolarization dispersion and autonomic nerve activity in a canine experimental acute myocardial infarction model. Heart Rhythm. 2014;11(1):110‐118. 24120873 10.1016/j.hrthm.2013.10.022 PMC4078249

[17] Piccirillo G , Moscucci F , Claudia DI . Time‐ and frequency‐domain analysis of repolarization phase during recovery from exercise in healthy subjects. Pacing Clin Electrophysiol. 2020;43(10):1096‐1103. 32789871 10.1111/pace.14038

[18] Moriyama H , Kohno T , Kohsaka S , et al. Length of hospital stay and its impact on subsequent early readmission in patients with acute heart failure: a report from the WET‐HF Registry. Heart Vessels. 2019;34(11):1777‐1788. 31134379 10.1007/s00380-019-01432-y

[19] Charles A , José MDD . Tpeak‐Tend interval as a marker of arrhythmic risk. Heart Rhythm. 2019;16(6):954‐955. 30660560 10.1016/j.hrthm.2019.01.017 PMC6545252

[20] Marek Malik HV , Huikuri F , Lombardi G , Schmidt RL , Verrier M . Zabel e‐Rhythm Group of EHRA is the T _peak_‐T _end_ interval as a measure of repolarization heterogeneity dead or just seriously wounded? Heart Rhythm. 2019;16(6):952‐953. 30660561 10.1016/j.hrthm.2019.01.015

[21] Tse G , Gong M , Wong WT , et al. The T _peak_ ‐ T _end_ interval as an electrocardiographic risk marker of arrhythmic and mortality outcomes: a systematic review and meta‐analysis. Heart Rhythm. 2017;14(8):1131‐1137. 28552749 10.1016/j.hrthm.2017.05.031

[22] Ann‐Kathrin R , Lugenbiel P , Schweizer PA , Katus HA . Dierk Thomas role of ion channels in heart failure and channelopathies. Biophys Rev. 2018;10(4):1097‐1106. 30019205 10.1007/s12551-018-0442-3 PMC6082303

[23] Piccirillo G , Magrì D , Ogawa M , et al. Autonomic nervous system activity measured directly and QT interval variability in normal and pacing‐induced tachycardia heart failure dogs. J Am Coll Cardiol. 2009;54(9):840‐850. 19695465 10.1016/j.jacc.2009.06.008 PMC2756779

[24] Piccirillo G , Moscucci F , Pofi R , et al. Changes in left ventricular repolarization after short‐term testosterone replacement therapy in hypogonadal males. J Endocrinol Invest. 2019;42(9):1051‐1065. 30838540 10.1007/s40618-019-01026-5 PMC6692303

[25] McDonagh TA , Metra M , Adamo M , et al. ESC Scientific Document Group 2021 ESC Guidelines for the diagnosis and treatment of acute and chronic heart failure. Eur Heart J. 2021;42(36):3599‐3726. 34447992 10.1093/eurheartj/ehab368

[26] Eapen ZJ , Reed SD , Li Y , et al. Do countries or hospitals with longer hospital stays for acute heart failure have lower readmission rates? Findings from ASCEND‐HF. Circ Heart Fail. 2013;6(4):727‐732. 23770519 10.1161/CIRCHEARTFAILURE.112.000265

